# The Current Understanding of MicroRNA’s Therapeutic, Diagnostic, and Prognostic Role in Chordomas: A Review of the Literature

**DOI:** 10.7759/cureus.3772

**Published:** 2018-12-24

**Authors:** Paul J Choi, Rod J Oskouian, R. Shane Tubbs

**Affiliations:** 1 Surgery, Seattle Science Foundation, Seattle, USA; 2 Neurosurgery, Swedish Neuroscience Institute, Seattle, USA; 3 Neurosurgery, Seattle Science Foundation, Seattle, USA

**Keywords:** sacral chordoma, microrna, chemoradiotherapy resistance, biological target, prognosis, differential expression, mirna profiling

## Abstract

Chordomas are primary low-grade bone tumors derived from the embryonic notochord that make up less than 5% of all osseous malignancies and commonly affect the spine at its vertebral body and at its two ends i.e., skull base and the sacrum. Although histologically defined to be low-grade, chordoma is locally destructive, metastatic, and has a serious recurrence rate, which all contribute to the dismal median survival rate of six years. Its locally destructive nature places the adjacent vital neurovascular structures at risk, making an en-bloc resection a challenge. This tumor is also known to show high resistance to currently available chemoradiotherapy, although the benefit of proton beam therapy for skull base chordoma has been demonstrated. There is an additional need to focus our attention on investigating the molecular biology of this chemoradiotherapy-resistant tumor to develop a more targeted therapy, which has additional diagnostic and prognostic values. In this paper, we discuss the therapeutic, diagnostic, and prognostic role of microRNAs (miRNAs) in chordomas.

## Introduction and background

Chordomas are primary bone tumors that make up less than 5% of all osseous malignancies and commonly affect the spine at its vertebral body and at its two ends i.e., the skull base and the sacrum [1-3]. It is a low-grade tumor derived from the embryonic notochord [1-2,4-5]. Although histologically defined to be low-grade, chordoma is locally destructive and metastatic in up to 19% of all chordoma cases, according to Bydon et al. [2], and has a serious recurrence rate, which all contribute to the dismal median survival rate of six years [2-3,5]. The treatment modality of choice is an en-bloc resection [1-2]. It has been suggested that neoadjuvant radiotherapy may have a role in preventing potential hematogenous spread of the tumor during surgical manipulation [2]. However, this tumor is known to show high resistance to currently available chemoradiotherapy [1,3,5], although the effectiveness of proton beam therapy for skull base chordomas has been demonstrated [6-7]. The literature even claims that conventional chemotherapy has no role in treating this disease [3,8]. Its locally destructive characteristic places the adjacent vital neurovascular structures at risk, making an en-bloc resection a challenge [2,5]. Although recent advancements in surgical techniques have made a complete resection with negative margins achievable, the disappointing recurrence rate persists [2]. Hence, there is an additional need to focus our attention on investigating the molecular biology of this relatively chemoradiotherapy-resistant tumor to develop a more targeted therapy that may carry additional diagnostic and prognostic values.

## Review

miRNA expression in chordomas

The recent arrival of better understanding of microRNA's (miRNA) molecular roles in cell proliferation has shed light upon improved knowledge in the pathogenesis of many solid and hematologic malignancies such as lung, breast, and prostate cancers, and chronic lymphocytic leukemia [2-3,5,9]. miRNA is a small (20-30 nucleotides in length), non-coding, single-stranded ribonucleic acid (RNA) molecule [3-5], which suppresses messenger RNA (mRNA) transcription and disrupts subsequent gene regulation in eukaryotic cells [2,4]. By doing so, it can either induce tumorigenesis or trigger tumor suppression depending on the part of the human genome it occupies [2,9]. miRNA tends to be located in a genetically unstable part of the chromosome and, hence, are often dragged into a molecular cascade that leads to chromosomal abnormalities and oncogenic mutations [9-11]. Further, miRNAs are known to control many genes involved in cell proliferation and they can be an inhibitor of one of such genes and a stimulator of another simultaneously [10].

Due to the rarity of the disease, miRNA’s role in chordoma’s pathogenesis and its potential diagnostic and prognostic values have been seldom explored in the literature [12]. However, existing data suggest that chordomas demonstrate a significantly different expression of miRNA compared to the control [2]. Once a miRNA that is involved in chordomagenesis is identified, either a highly specific miRNA inhibitor or recombinant miRNA can be synthesized to neutralize oncogenesis [10-11]. This is unlike conventional chemotherapy, which often targets gain-of-function mutations [10-11]. This unusual mechanism may have a role in the non-surgical management of chordoma [10].

miRNA downregulation in chordomas

To date, there are no reports that focus mainly on upregulated miRNA in the pathogenesis of chordoma [11]. Recent studies report that miRNA hypermethylation (inactivation) is associated with metastasis and recurrence of cancer. For instance, miRNA 9 family downregulation has been described to be linked to metastasis and recurrence of gastric adenocarcinoma, renal cell carcinoma, and colorectal carcinoma [11]. This is in-line with chordoma’s metastatic and extremely recurrent features that have been described for many decades.

Met proto-oncogene, which is highly associated with miRNA 1 and described by Duan et al. to be downregulated in 93.7% of chordomas, was overexpressed in 94.4% of all chordomas [1-2,5,12-13]. A more recent study by Duan et al. reports that miRNA 1 is not only a potential therapeutic target, but that it is also a prognostic marker i.e. a lower level of the miRNA is associated with poorer prognosis [13]. Furthermore, in-vitro and in-vivo studies have confirmed this particular miRNA’s anti-cancer activity [13]. miRNA 34a and 608 have also been pronounced as tumor suppressors, which are downregulated in chordoma [1,14]. Zhang et al. explain that these two miRNAs regulate multiple oncogenes including the Met gene [14]. Kuang et al. report that miRNA 10a and 125a are downregulated in skull base chordoma [15]. miRNA 10a and 125a are antitumor miRNAs, which are suppressed by adenosine deaminases acting on RNA (ADAR) gene, which is overexpressed in skull base chordoma [15].

Bayrak et al. and Gulluoglu et al. suggest that miRNA 31-3p, 148a, and 222-3p impose a pro-apoptotic effect on chordoma by regulating Met and Radixin oncogenes, DNA methyltransferase (DNMT) pro-apoptotic gene, and c-KIT proto-oncogene respectively, subsequently inducing a cell cycle arrest at the S-phase and preventing progression to G2 phase [3-4,15-16]. The miRNAs are also expressed varyingly in different chordoma cell lines. For instance, miRNA 222-3p is downregulated in U-CH1 but upregulated in U-CH2 chordoma cell lines [3].

Besides the miRNAs described above, numerous others have been reported to be associated with the pathogenesis of chordoma. For example, Bayrak et al. report an additional 53 dysregulated miRNAs in skull base chordoma compared to the biology of normal nucleus pulposus, and Wei et al. demonstrate the chordomagenic effect of miRNA 219-5p expression and its association with poor prognosis by studying spinal and sacral chordomas [4,17].

miRNA as a potential diagnostic and prognostic marker of chordoma

Bayrak et al. and Gulluoglu et al. report that miRNA 140-3p upregulation is associated with the metastatic and recurrent characteristics of spinal chordomas, hence it is a poor prognostic marker [3-4,12]. miRNA 155 upregulation is also associated with poor prognosis of sacral chordoma, suggesting its potential use in targeting the highly recurrent and metastatic nature of chordomas [1,8]. Zou et al. reveal miRNA 1237-3p upregulation as an independent predictive measure of good prognosis of chordoma [1,18]. This is the “independent” “unknown function” of miRNA in oncogenesis described by Calin et al. [9].

Furthermore, there have been no studies specifically exploring the sacral type of chordoma. Bayrak et al. and Zou et al. look at skull base and spinal chordomas [4,12,18]. Wei et al. study both spinal and sacral chordomas [17]. Although miRNA 155 upregulation was studied to be associated with poorer prognosis of sacral chordoma by Osaka et al., an isolated study of the sacral chordoma’s molecular pathogenesis i.e., identification of certain miRNAs’ association with a specific oncogene, is lacking [8]. In addition, the sacral type has not yet shown to be responsive to proton beam therapy, unlike its skull base counterpart. Hence, there is a dire need to explore this type of tumor.

## Conclusions

The understanding of dysregulation of various miRNAs in chordoma can be used therapeutically and for early detection and prediction of the prognosis of the tumor. Further miRNA profiling studies will provide a better understanding of its role in chordomagenesis and potentially offer a foundation, upon which to develop a novel targeted therapy for this mainly chemoradiotherapy-resistant tumor. However, to the best of our knowledge, there have been no clinical trials to study miRNA as a potential therapeutic target for chordomas.
